# Diseases of Stark County, Ohio

**Published:** 1842-08

**Authors:** 


NOTICES OF THE WEATHER AND DISEASES IN THE WINTER OF 1841–2, AT PARIS, STARK COUNTY, OHIO, IN A LETTER TO THE SENIOR EDITOR FROM C. H. PRESTON, M. D.

"In order to fill my sheet I will give you a synopsis of the character of our past winter and its attendant diseases. As to the former, suffice it to say that it was somewhat remarkable for variableness—much rain, litttle snow, no sleighing, and the lake not frozen over. The prevailing winds were from the east and north east, and have continued during the spring months, and up to the present moment. Vegetation commenced nearly two weeks earlier than usual, but for the last four or five weeks the weather has been (frequently at least) disagreeably cool. Agricultural prospects are, however, quite promising.

Ours is ordinarily a very healthy region, there being few sources of miasm, and the face of the country generally somewhat undulating, with an abundance of pure running water—the spring and wells somewhat impregnated with lime. Our diseases, then, are generally such as are engendered by sudden vicissitudes of weather, which are frequent and sudden, owing in part to our position, in common with most of the northern part of the state, in a region where the influences of the northern lakes, the Allegheny mountains and the Mississippi valley react. These diseases consist of pneumonias, and other inflammatory affections, with now and then an epidemic of typhoid pneumonia, scarlatina, dysentery, &c. Bilious fevers are not prevalent to a great extent, or of a severe grade among us, excepting on the Ohio canal. But to the epidemic of the last winter. The season was as healthy as usual till the month of December, when more than the usual number of cases of pneumonitis and acute hepatitis occurred, with other inflammatory affections, the result of exposure or the sudden changes which began to visit us thick and fast. About the first of January the scarlet fever made its appearance both in this and in several of the adjoining counties at the same time. Its appearance and spread were evidently independent of contagion, no precaution or remoteness affording any security. As far as my observations extended it was generally of the congestive or congesto-inflammatory grade, and proved peculiarly unmanageable. A number of cases, evidently the result of the same great epidemic constitution or influence, occurred, in which the little patients were taken off in from 16 to 24 hours after the first appearance of indisposition. In these cases the nervous system, and particularly the brain, sustained the burden of the shock.

A pale or purplish appearance of the surface, shrunken features, frequent, feeble or depressed pulse, frequent vomiting of a greenish or purplish fluid, absence of expression in the eyes, the conjunctiva injected, cool feet and preternaturally warm head, clammy, slightly furred tongue, and before death, convulsions; these cases thus rapidly fatal—the patient being sometimes dead, sometimes in articulo mortis when I was called in—could not be termed scarlatina proper, as there was no eruption or sore throat, the essential characteristics of that disease; but from the identity of the general symptoms I have no doubt of the identity of their general nature, one great constitutional predisposition acting as the moulding and controlling agent over them all.

I need not trouble you with the treatment, for in these cases it was of little avail. Warm baths, small portions of cal. and ipecac., stimulating injections, warm drinks (in genuine congestion), cold to the head, sinapisms to the feet, blisters between the shoulders, stimulating cataplasms to the abdomen—in short, such means as I deemed proper to divert the circulation from the brain, internal large vessels and other vital organs, and to equalise the circulation. Cupping the temples under similar circumstances in scarlatina was sometimes found very beneficial in relieving the head.

As to the scarlatina proper, I observed that it was mostly of the congestive or congesto-inflammatory grade. The treatment, of course, would not differ much in its general principles from what I have detailed above. In fact, very little medication was found necessary or of much avail—good nursing being of equal importance.

The disease in this vicinity was not as much inclined to result in severe ulceration of the throat, ears, &c., as I have frequently observed it to be, though several such cases occurred. The shock was sustained by the lungs, brain, and general system. The proportion of deaths generally was somewhat greater than in common epidemics of this disease. It continued with little abatement through the months of January and February, and was confined principally to children. During the latter part of the epidemic the measles accompanied it, and became quite prevalent. They were observed to be more obstinate than usual, owing to the general depression of the vital organs. It was difficult, in many instances, to obviate their tendency to settle on the lungs in the form of congestion or inflammation; and although there was no death from them in my practice, they proved extensively fatal in some neighborhoods not far distant, many adults as well as children being carried off by them. Blood-letting proved beneficial in most cases of this disease where an inflammatory tendency existed, a modification that I thought did not generally obtain, very few patiently as a general rule bearing the remedy to much extent.

At this time, and for some weeks past, the sunshine of health is dispensing its welcome influence over our country; and the afflictions of the past are giving way to the interests and engagements of the present."

*June 8th*, 1842.

